# Attitudes toward integrative paediatrics: a national survey among youth health are physicians in the Netherlands

**DOI:** 10.1186/1472-6882-12-4

**Published:** 2012-01-16

**Authors:** Miek C Jong, Marja van Vliet, Susan Huttenhuis, Désirée van der Veer, Silvia van den Heijkant

**Affiliations:** 1 Department Healthcare and Nutrition, Louis Bolk Institute, Driebergen, the Netherlands; 2 Department Health Sciences, Mid Sweden University, Sundsvall, Sweden; 3 Community Healthcare centre Twente (GGD), Enschede, the Netherlands; 4 Department of Public and Occupational Health, EMGO + Institute for Health and Care Research, VU University medical centre, Amsterdam, the Netherlands

**Keywords:** National survey, Integrative Paediatrics, Attitudes, Youth Healthcare

## Abstract

**Background:**

Integrative Medicine (IM) is an emerging field in paediatrics, especially in the USA. The purpose of the present study was to assess the attitudes and beliefs of Youth Health Care (YHC) physicians in the Netherlands toward IM in paediatrics.

**Methods:**

In October 2010, a link to an anonymous, self-reporting, 30-item web-based questionnaire was mailed to all members of the Dutch Organisation of YHC physicians. The questionnaire included questions on familiarity with IM, attitudes towards Integrative Paediatrics (IP), use and knowledge of Complementary and Alternative Medicine (CAM), demographic and practice characteristics.

**Results:**

A total of 276 YHC physicians (response rate of 27%) responded to the survey. Of the respondents, 52% was familiar with IM and 56% had used some kind of CAM therapy during the past 2 years, of which self-medicated herbal and/or homeopathic remedies (61%) and supplements (50%) were most frequently mentioned. Most of the YHC physicians (62%) seldom asked parents of clients about CAM use. One third of the YHC physicians recommended CAM to their clients. In general, about 50% or more of the respondents had little knowledge of CAM therapies. Predictors for a positive attitude towards IP were familiarity with IM, own CAM use, asking their clients about CAM use and practising one or more forms of CAM therapy. Logistic regression analysis showed that the following factors were associated with a higher recommendation to CAM therapies: own CAM use (odds ratio (OR) = 3.8; 95% confidence interval (CI) = 2.1-6.9, *p *= 0.001) and practising CAM (OR 4.4; 95% CI = 1.6-11.7, *p *= 0.003).

**Conclusions:**

In general Dutch YHC physicians have a relative positive attitude towards IP; more than half of the respondents used one or more forms of CAM and one third recommended CAM therapies. However, the majority of YHC physicians did not ask their clients about CAM use and seemed to have a lack of knowledge regarding CAM.

## Background

During the last decade, Integrative Medicine (IM) is an emerging field in health care, especially in the USA. IM is perceived as a health care approach that is client centred and healing oriented. It embraces conventional therapies as well as Complementary and Alternative Medicine (CAM) [1]. In IM, particular attention is given to: 1. The health care provider-client relationship; 2. Prevention strategies; 3. Healing environment and 4. Making use of all appropriate therapeutic treatments, including CAM [2,3]. In 2000 the Consortium of Academic Health Centres on IM was established, consisting nowadays of 47 academic health care centres [4]. IM has also extended to the field of paediatrics [5]. In 2004 the Integrative Paediatrics (IP) Council was formed to develop and support programs and research in the area of integrative paediatrics [6]. In the Netherlands, an Integrative Paediatrics program was established at the Slotervaart Hospital (Amsterdam) [7]. In 2009, about 9.2% of the general population in the Netherlands received one form of CAM therapy [8]. In children visiting a general paediatric clinic, CAM use was reported to be much higher (33%) [9]. Despite the frequent CAM use by children, the majority of parents do not report CAM use to their paediatrician [9]. In line with these findings it has been reported that 62% of the paediatricians do not discuss CAM with their clients, partly since they have little knowledge on this subject [10]. However, the paediatrician is not the only health care provider for children. In the Netherlands, every new born is automatically registered by a Youth Health Care (YHC) organisation. At those YHC centres, specialized YHC physicians are focussed on prevention of diseases, monitoring of growth/development and immunization of children in the age of 0-19 years [11]. During the first 4 years of a child's life, there are 15 checkups by the YHC physician. In the Netherlands, the YHC program is free of charge, voluntary and the attendance rate is very high (95%) [12]. Each country within the European Union organizes YHC in a different way. However, basic activities such as immunization, monitoring, detection and screening and a clear separation of preventive from curative services are covered by YHC in most European countries [13]. The role of the YHC physician differs from that of the family physician and paediatrician in that the latter focuses primarily on the treatment of diseases in children. Among YHC physicians, there is a clear focus on prevention, lifestyle and environment. Although these aspects are also underlined in IP, up till now no data are available on the attitudes and knowledge of YHC physicians towards this integrative approach. The purpose of the present study was therefore to assess Dutch YHC physician's attitudes and beliefs on IP, with specific attention to their experiences with and knowledge on CAM therapies.

## Methods

### Questionnaire

Data were collected through a structured, anonymous, self-reporting questionnaire. This questionnaire was modified from the questionnaires as previously used in surveys among Dutch healthcare professionals and managers [14] and Dutch paediatricians [10]. Before distribution, the questionnaire was piloted among five YHC physicians after which modifications were made. The final survey consisted of 30 questions on IM and CAM. First attitudes on CAM were surveyed making use of different statements. Furthermore, physicians were asked for own CAM use. A list of the most common forms of CAM in the Netherlands was provided: phytotherapy, dietary supplements, Chinese medicine, homeopathy, body-mind techniques (including yoga, hypnosis, and meditation), energy medicine, bioelectrical therapies, manipulative therapies, and anthroposophical medicine. Physicians were given the opportunity to report additional CAM therapies. Other aspects surveyed were communication with clients regarding CAM therapies, referral to CAM therapies and knowledge of CAM. The survey also contained 3 clinical vignettes: 1. A 3-year old boy with sleeping disorder; 2. A 9-year old boy with attention deficit hyperactivity disorder (ADHD) and 3. A 15-year old girl with chronic abdominal pain. Physicians were asked to indicate which CAM therapies they regularly, occasionally or never would recommend in each of these situations. The physicians were than asked about their knowledge of and attitudes towards IM. In the last section of the survey, nine questions on demographic characteristics (age, sex, location, organisation, specialisation and work experience) were listed.

### National Survey

In October 2010 all members of the Dutch Organisation of Youth Health Care physicians (the AJN) were invited by mail to fill out this web-based questionnaire. The questionnaire was closed after six weeks. In order to increase the response rate, the survey was posted on the website of the AJN, announced in their monthly newsletter and at an AJN member meeting in November 2010, YHC physicians were reminded to complete the survey. The board of the AJN approved the study.

### Statistical Analyses

The analysis is based on the full and complete dataset. The web-based questionnaire was designed as that each question had to be fully answered before continuing to the next question and consecutive completion of the questionnaire. Descriptive statistics were used to summarize the demographics of the respondents and their responses to the questions. Chi-square analyses were used for bivariate comparisons. Univariate and multiple logistic regressions for associations between YHC physician characteristics and their attitudes and beliefs on IM and CAM (significance level (*p *< 0.05). Data were analysed using SPSS 15.0.

## Results

### Characteristics of YHC physicians

A total of 1013 AJN members were approached by email. A total of 276 YHC physicians (27%) responded to the survey. The majority of respondents were female (92%) and practiced at a community health care centre (GGD) (59%) (see Table 1). A little more than half of the respondents (51%) worked with children between 0-4 years of age. The highest percentage of respondents with respect to age was found in the group of 46-55 years (34%). With respect to geographical location, the highest percentage of respondents was found in the conurbation (27%) of the Netherlands (e.g. Amsterdam, Rotterdam, Utrecht and The Hague). Furthermore, 38 % of the respondents were YHC physicians with qualifications of the KNMG (Royal Dutch Physician Association), 30% were YHC physicians with training and management in social medicine, 11% of respondents were in training as a KNMG YHC physician and 12% of the respondents worked as a physician in YHC, but had no specific YHC qualifications.

**Table 1:** Characteristics of respondents

Characteristics	Percentage* N = 276
**Gender**	
Male	8 %
Female	92 %

**Age (years)**	
< 35	16 %
36-45	24 %
46-55	34 %
56-65	23 %
>65	2 %

**Experience (years)**	
< 2	3 %
2-10	25 %
>10	72 %

**Region**	
North	18 %
Mid	24 %
Conurbation	27 %
South	20 %
East	15 %

**Organizations**	
Community health care centre (GGD)	59 %
Homecare organisation	35 %
Hospital	1 %
Other	13 %

**Age of children (years) they work with**	
0-4	51 %
4-19	33 %
0-19	11 %
0-12	4 %
12-19	1 %

*Percentile may add to less or more than 100% due to rounding.

### Attitudes and familiarity with Integrative Medicine (IM)

Of the YHC physicians, 52% were familiar with IM. A total of 44% of the respondents regarded IM of little importance as a new vision on healthcare and 40% had a neutral opinion towards IM. Further data on attitudes of YHC physicians towards three major components of IM are shown in Table 2. With respect to the first component of IM, the health care provider-client relationship, the majority (60%) of respondents believed that clients want a coaching physician. Almost all YHC physicians (99%) were of the opinion that a doctor should be able to use motivational techniques in consultation. Furthermore, 87% agreed that a physician should inform the client about all possible treatments, leaving the choice of treatment with the client. In addition, 43% agreed that the computer had an excessive role in their consultations. With respect to the second component of IM, prevention strategies, the majority of the respondents (73%) believed that self-management means that the client is complaint to the treatment given. One third of respondents confirmed that it is the responsibility of the client to acquire extra information on the disease or treatment. Furthermore, 56% did consider that self-management means that the client has to change his/her lifestyle. With respect to the third component of IM, healing environment, almost all respondents believed that a pleasant environment influences the client healing positively (92%) and was of the opinion that a pleasant environment has a positive effect on the functioning of a child and caregivers (98%).

**Table 2:** Respondents beliefs regarding IM

Respondents' beliefs regarding	(totally) agree	not agree/ not disagree	(totally) disagree
	Percentage* N = 276		
**... the health care provider-client relationship**			
• Clients don't want a coaching physician, they want a physician to decide what is best for them	12 %	28 %	60 %
• Each physician should be capable of applying motivational interviewing	99 %	1 %	1 %
• The physician should inform the client accurately about all possible treatments for the client to make his/her own choice	87 %	7 %	6 %
• The computer is overwhelmingly present during consultations	43 %	24 %	32 %
**... prevention strategies**			
• Self-management means the client is compliant to the treatment given	73 %	20 %	6 %
• The clients is responsible to acquire extra information concerning the diagnosis or treatment	33 %	28 %	39 %
• Self-management means that the client has to change his/her lifestyle	56 %	30 %	15 %
**... healing environment**			
• A pleasant environment influences the clients' healing positively	92 %	8 %	0 %
• A pleasant environment has a positive effect on the functioning of the child and caregivers	98 %	2 %	0 %
• The interaction between body, mind and environment is nothing less than a trend	6 %	9 %	83 %

*Percentile may add to less or more than 100% due to rounding.

### Attitudes, beliefs and knowledge on CAM

Since most controversy in IM is directed toward the fourth component of IM, the use of CAM, this component was addressed in more depth. In Table 3 it is shown that the majority (56%) of YHC physicians used some form of CAM themselves, two years prior to the survey. Most frequently cited were self-medicated herbal and/or homeopathic remedies (61%), dietary supplements (50%), manual therapies (32%) and mind-body therapies (24%). Almost two thirds (62%) of the YHC physicians seldom or never asked their clients whether they were using CAM. Only a small number (9%) practiced some kind of CAM themselves, mostly homeopathy (23%), anthroposophic therapy (20%) or mind-body therapy (17%). A substantial number of YHC physicians (34%) referred frequently/ occasionally to CAM. Most referrals were made to homeopaths (33%), manual therapists (32%), acupuncturists (10%), self-medicated homeopathic remedies and mind-body therapies (5%).

**Table 3:** Respondents' behaviour regarding CAM

Item	Percentage *N = 276*
Personal use of CAM	56%

Ask clients about CAM use	
Never	17 %
Seldom	45 %
25% of the clients	18 %
25-50% of the clients	9 %
75% of the clients	6 %
100% of the clients	4 %

Self practising CAM	
Yes	9 %
No	91 %

Refer to CAM practitioner	
Yes, frequently	2 %
Yes, occasionally	32 %
No	66 %

*Percentile may add to less or more than 100% due to rounding.

In Table 4 the beliefs of YHC physicians regarding CAM are shown. Most YHC physicians (77%) agreed that "the physician should inform the client about CAM if the client asks for it". In general, respondents were reluctant with respect to implementation of CAM within their own organisation. The majority (58%) thought their organisation should not advise CAM and 62% was afraid that CAM harms the reputation of their organisation. Most respondents had little concern with the safety of CAM, e.g. 50% thought that CAM does not negatively interfere with standard medical care and only 11% believed that CAM use risks additional side effects. With respects to the possible positive health effects of CAM therapies, only 28% believed that CAM enhances recovery and symptom relief (see Table 4). The general knowledge of YHC physicians with respect to CAM is shown in Table 5. The CAM therapies of which YHC physicians think they know most about is probiotics (56%), followed by homeopathy (47%), dietary supplements (44%) and manual therapies (43%). With respect to the need for more information on CAM, YHC physicians had interest in courses on probiotics, manual therapies, dietary supplements and homeopathy (Table 5).

**Table 4:** Respondents beliefs towards CAM

**Respondents' beliefs regarding**	**(totally) agree**	**not agree/** **not disagree/** **no opinion**	**(totally) disagree**
	**Percentage** *N = 276*		
• My organisation shouldn't offer CAM to clients	58 %	15 %	26 %
• CAM can harm your (organisation) reputation	62 %	15 %	23 %
• CAM use interferes negatively with standard medical care	15 %	35 %	50 %
• CAM use enhances recovery and symptom relief	28 %	43 %	29 %
• CAM use risks additional side effects	11 %	46 %	44 %
• CAM use decreases the overall health care costs	23 %	31 %	46 %
• The physician should inform the client about CAM when the client ask for it	77 %	11 %	13 %

*Percentile may add to less or more than 100% due to rounding.

**Table 5:** Respondents knowledge regarding CAM

	**Knowledge level**			**Need for more education**	
	**excellent/moderate**	**little**	**nothing**	**yes**	**no**
**CAM therapy**	Percentage* N = 276				
Probiotics	56 %	31 %	14 %	50 %	50 %
Homeopathy	47 %	40 %	14 %	39 %	61 %
Dietary supplements	44 %	37 %	19 %	46 %	54 %
Manual therapies	43 %	40 %	17 %	48 %	52 %
Mind-body therapies	25 %	33 %	42 %	36 %	64 %
Chinese medicine (incl. acupuncture)	26 %	39 %	35 %	36 %	64 %
Anthroposophy	23 %	36 %	42 %	34 %	67 %
Herbal remedies	20 %	36 %	44 %	35 %	65 %
Naturopathy	19 %	32 %	50 %	33 %	67 %
Energetic therapies	12 %	26 %	62 %	27 %	74 %
Bioelectric therapies	12 %	28 %	60 %	26 %	74 %

*Percentile may add to less or more than 100% due to rounding.

### Recommendations to CAM

About half of the YHC physicians recommended a CAM therapy in the three-presented clinical cases (see Table 6). In the case of the 3-year-old boy with sleeping disorders, over 49% of the YHC physicians would recommend some kind of dietary advice (48%), followed by manual therapies (30%) or homeopathy (30%). Other CAM therapies recommended were mind-body therapies (16%), herbal remedies (15%) or dietary supplements (12%). For the 9-years-old boy with ADHD, YHC physicians recommended mostly dietary advice (49%). In the last case, presenting the 15-year-old girl with chronic abdominal pain, 74% of respondent would recommend dietary advice. Other CAM therapies recommended were mind-body therapy (40%), homeopathy (27%), manual therapy (26%) and dietary supplements (21%).

**Table 6:** Respondents recommendations to CAM therapies for three clinical cases

	Percentage* N = 276		
**A three year old boy with sleeping disabilities**	**usually**	**occasionaly**	**never**
Dietary advice	16 %	33 %	52 %
Herbal remedies	4 %	10 %	87 %
Mind-body therapies	3 %	13 %	84 %
Homeopathy	2 %	27 %	71 %
Massage and other manual therapies	2 %	28 %	70 %
Anthroposophy	2 %	9 %	90 %
Dietary supplements	1 %	11 %	88 %
Naturopathy	1 %	10 %	99 %
Acupuncture	1 %	5 %	95 %
**A nine year old boy with ADHD**	**usually**	**occasionaly**	**never**
Dietary advice	11 %	38 %	51 %
Dietary supplements	5 %	18 %	78 %
Mind-body therapies	4 %	21 %	76 %
Neurofeedback	3 %	26 %	71 %
Massage and other manual therapies	2 %	17 %	81 %
Anthroposophy	2 %	7 %	92 %
Homeopathy	2 %	17 %	82 %
Naturopathy	2 %	7 %	92 %
Other: Bachbloesemtherapy	2 %	5 %	94 %
Acupuncture	1 %	6 %	94 %
**A fifteen year old girl with chronic abdominal pain**	**usually**	**occasionaly**	**never**
Dietary advice	48 %	27 %	26 %
Mind-body therapy	8 %	32 %	60 %
Massage and other manual therapies	3 %	23 %	74 %
Dietary supplements	3 %	18 %	79 %
Homeopathy	3 %	24 %	73 %
Acupuncture	2 %	11 %	87 %
Naturopathy	2 %	10 %	87 %
Energetic therapies	2 %	9 %	89 %
Herbal remedies	1 %	11 %	89 %

*Percentile may add to less or more than 100% due to rounding.

### Characteristics associated with favourable attitudes towards IM and CAM

There was a significant association between YHC physicians who were familiar with IM and their opinion towards IM as an important healthcare vision (*p *< 0.001). Furthermore, there was a significant association between YHC physicians who recommended CAM therapies to their clients and the YHC physicians that practised a CAM therapy (*p *< 0.001). Characteristics of importance for YHC physicians practising a CAM therapy were if they used some kind of CAM therapy themselves (*p *< 0.001) and if they were familiar with IM (*p *< 0.001). A positive attitude of YHC physicians toward CAM was not found to be dependent of age, work experience, or age of children they worked with. When all the variables were entered into a logistic regression analysis, the following factors were associated with a higher recommendation to CAM: own CAM use (OR = 3.8; 95% CI = 2.1-6.9, *p *= 0.001) and practising CAM (OR 4.4; 95% CI = 1.6-11.7, *p *= 0.003) (Table 7). Logistic regression analysis also demonstrated that YHC physicians asked clients more frequently about CAM when they had a high knowledge level compared to a low knowledge level of CAM (OR = 4.2; 95% CI = 1.9-9.6, *p *= 0.001) (Table 7).

**Table 7:** Predicting factors associated with favourable attitudes toward IM

Recommending CAM*	**OR (95% CI)**	***p-value***
Own CAM use (no)	1	
Own CAM use (yes)	3.8 (2.1 - 6.9)	*0.001*
Practising CAM (no)	1	
Practising CAM (yes)	4.4 (1.6 - 11.7)	*0.003*
Asking clients about CAM**	**OR (95% CI)**	***p-value***
Low knowledge	1	
Moderate knowledge	1.8 (0.9 - 3.7)	*0.110*
High knowledge	4.2 (1.9 - 9.6)	*0.001*

*Results from logistic regression analysis, adjusted for gender, age, CAM knowledge, age of children they work with and years of experience. **Results from logistic regression analysis, adjusted for gender, age, age of children they work with, years of experience, own use of CAM and own practice of CAM. OR, odds ratio; CI, confidence interval.

## Discussion

In the current survey, most Dutch YHC physicians were familiar with the concept of IM. The majority of the respondents regarded the three major components of IM, e.g. health care provider-client relationship, prevention strategies and the healing environment of importance to their daily practice. The fact that most YHC physicians were familiar with these three components of IM, may explain their rather neutral or negative opinion to regard IM as a new innovation in healthcare. Overall, determinants of a positive attitude toward IM were familiarity with IM, self-practising of CAM, self-use of CAM and high knowledge level of CAM. Remarkably, the use of CAM by YHC physicians themselves was relatively high (56% of respondents), much higher than the CAM use recently reported for paediatricians (39%) [10]. Despite their high rate of self-medicated herbal and/or homeopathic medications, the majority (63%) of YHC physicians did not discuss CAM use with parents or children. A survey among parents of children visiting an outpatient paediatric clinic, demonstrated that there is a high need under parents to discuss CAM treatment options with their physician [9]. Thus from a clients perspective, it should be recommended that every physician asks about CAM use as part of their regular medical examination. Although most YHC physicians do not discuss CAM use with their clients, they do see the need to accurately inform the client about all possible preventive interventions, including those that CAM has to offer. A possible explanation why YHC physicians do not routinely talk to clients about CAM may be that at present CAM is not part of their standard screening and monitoring and that they work under enormous time restraints.

Of the YHC physicians' one third recommended CAM therapies to their clients. The CAM practitioners most referred to were homeopaths, manual therapists and acupuncturists. In the three clinical cases, dietary advice was mostly recommended. Many YCH physicians commented at the end of the survey that they did not regard dietary advice as a CAM therapy. CAM is defined as a group of diverse medical and health care systems, practices, and products that are not generally considered part of conventional medicine [15]. Since at present, dietary advice is still not part of routine every day care provided by family physicians and paediatricians, it was decided to include dietary advice and dietary supplements in the survey as part of CAM, in line with previous surveys among paediatricians in the USA [16,17].

YHC physicians exhibited most knowledge on probiotics, homeopathy and dietary supplements. The high knowledge level on homeopathy could be explained by the fact that during their residency, YHC physicians follow an introductory course on homeopathy. Another explanation may be that self-care homeopathic remedies are frequently used by small children [9] and that parents may mention this more often to YHC physicians than other CAM therapies. In general, YHC physicians were not so familiar with acupuncture or anthroposophy. Furthermore, only one quarter of the respondents reported to have knowledge on mind-body therapies. A recent survey showed a high interest among paediatricians to refer their clients to mind-body therapies [10]. Apparently mind-body therapies are not yet embedded in YHC. An interesting finding is that YHC physicians would like more training on subjects that they actually report to have the highest knowledge level on. About half of the respondents wanted additional training about probiotics, nutritional supplements and manual therapies. This indicated that YHC physicians are open to learn more about CAM therapies.

YHC physicians had little concern with safety aspects of CAM therapies. The majority was of the opinion that CAM therapies do not cause additional side effects nor interfere negatively with conventional medical care. Compared with surveys among paediatricians in the USA, these findings are very different. There, most of the health care professionals shared the opinion that CAM therapy may cause additional side effects and can be harmful [16,17]. A recent pharmacoviligance study in Australia has demonstrated that CAM use can be associated with serious adverse event [18]. Therefore, it is of importance for YHC physicians to be informed about CAM therapies so that they are also able to address safety issues of CAM therapies in communication with clients.

YHC physicians were very outspoken with respect to practising of CAM within their own community healthcare centre. More than half of the respondents thought that it will have a negative impact on the name of their organisation and their reputation if they advise CAM and that their organisation should not even advise CAM. This attitude can be explained by the fact that CAM does not fit into the concepts of their organisation. Another explanation can be the lively discussions in the Royal Dutch Association of Healthcare on the subject of CAM between advocates and opponents. Outside the organisation YHC physicians seem to have a relatively positive toward IM and CAM. The majority uses some form of CAM themselves, more than one third recommend CAM therapies to their clients and some practise one or more forms of CAM therapy.

This national survey among YHC physicians found that overall respondents have a relatively positive attitude towards IP, including CAM therapies. However, the present study also has its limitations. The percentage of respondents, a little over a quarter (27%), makes it insufficient to draw firm conclusions about the fact that the positive attitude towards IP counts for all YHC physicians in the Netherlands. There is a potential for response bias. Those who participated may have had an interest in CAM and were therefore more willing to invest time in filling out the questionnaire. Upon the assumption that all non-respondents to the present survey were anti-CAM, the percentage of YHC physicians referring clients to CAM therapies would drop to 9% and the number using CAM themselves to 16%. This assumption, however, does not seem realistic since a large survey among patients in the Netherlands showed that healthcare professionals react neutral (54%) or positive (41%) to disclosure of CAM use [19]. Furthermore, the average characteristics of respondents in the present study were very similar to another, non-CAM related published survey among members of the AJN [20]. It should be noted that in the Netherlands, YHC physicians are mostly women, leaving male YHC physicians a minority (about 8%). Within Europe, Belgium and the Netherlands represent particular examples where YHC is provided by specialized YHC physicians. In other European countries the paediatrician and/or family physician play a central role in providing YHC [13]. To our knowledge, the present study is the first one to assess the attitudes of health care providers about IP and CAM in YHC specifically. Therefore, it is difficult to translate the current findings to other European countries and the USA. However, world-wide several studies have reported on the positive attitudes of paediatricians towards CAM [10,15,16,21], suggesting that there is openness to further communicate and educate about possible implementation of IP healthcare programs in child health care.

## Conclusions

Overall, Dutch YHC physicians have a relatively positive attitude towards IP with about one third recommending patients to CAM therapies. A clear distinction could be made between where CAM therapies are practiced: i.e. there seemed to be more openness toward own CAM use and CAM practising than to implementation of CAM within their YHC organization. Although the majority of YHC physicians still did not ask patients about CAM use, the present survey may further contribute to their awareness of CAM and the necessity to discuss or provide information on CAM to the parents of their clients.

## Authors' contributions

MJ, SH, DvdV and SvdH developed and piloted the survey. SH and MvV performed the analysis. MJ wrote the manuscript. All authors read and approved the final version of the manuscript.

## Competing interests

The authors declare that they have no competing interests.

## Pre-publication history

The pre-publication history for this paper can be accessed here:

http://www.biomedcentral.com/1472-6882/12/4/prepub
